# Diffuse vulvar papules in a patient with Crohn’s disease

**DOI:** 10.1016/j.jdcr.2025.01.017

**Published:** 2025-02-05

**Authors:** Katlyn M. Smaha, Kaitlyn J. Blanchard, Matthew R. Powell, Loretta S. Davis

**Affiliations:** a Medical College of Georgia at Augusta University, Augusta, Georgia; b Department of Dermatology, Medical College of Georgia at Augusta University, Augusta, Georgia; c Department of Pathology, Medical College of Georgia at Augusta University, Augusta, Georgia

**Keywords:** Crohn’s disease, eruptive syringomas, inflammatory bowel disease, vulvar papules, vulvar syringomas

## History

**Fig 1:**
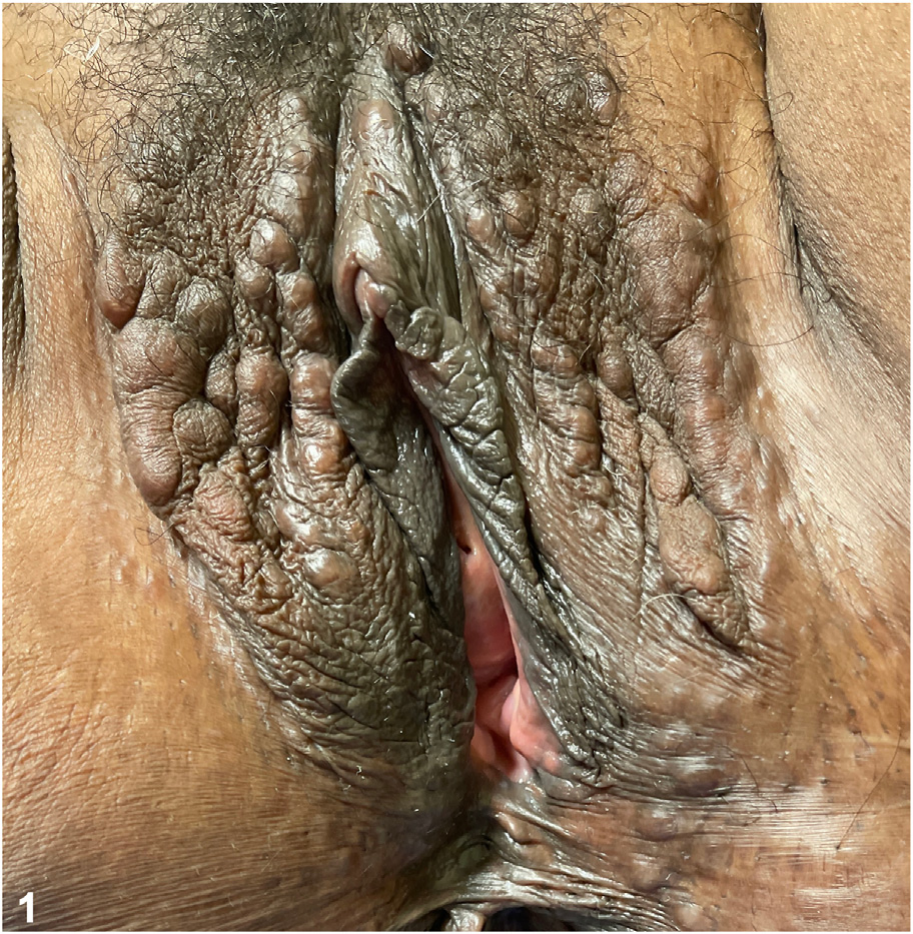
Clinical findings on exam.

**Fig 2:**
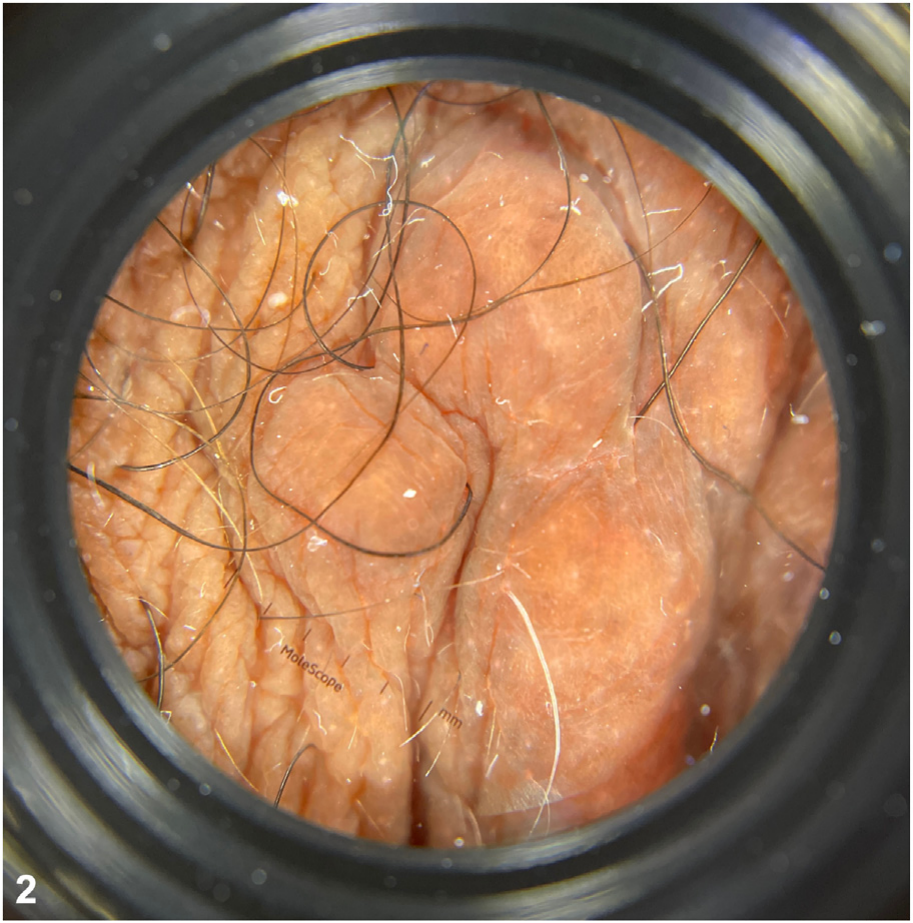
Dermoscopy findings on exam.

**Fig 3:**
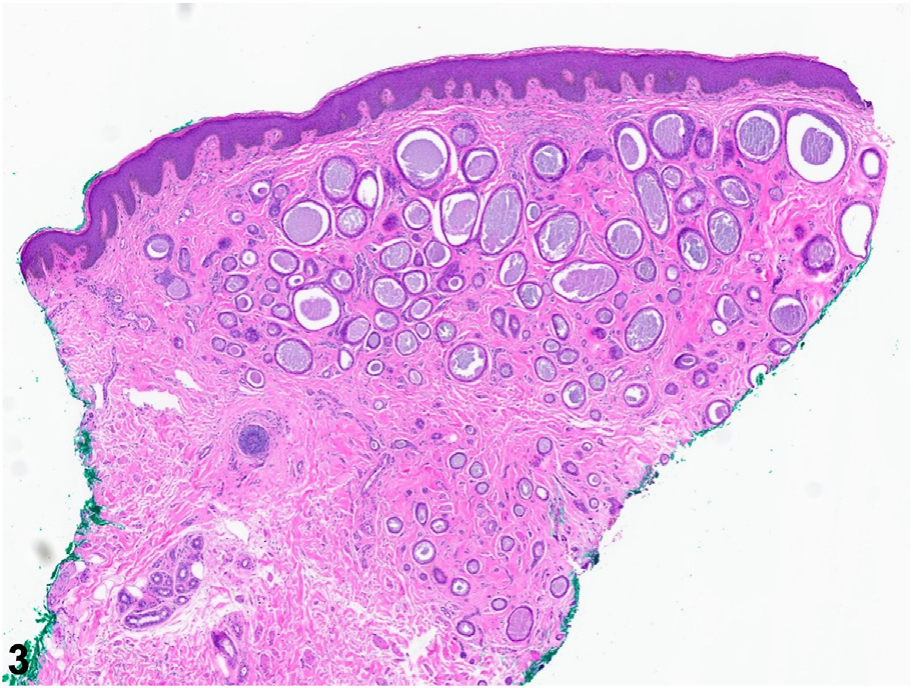
Hematoxylin and eosin (H&E) stain 4× showing punch biopsy findings.

A 55-year-old Black woman with a history of erythema nodosum and Crohn’s disease (CD) complained of bumps on her vulva and perineum of 3-4 months duration. The lesions were occasionally pruritic but not painful. In addition, she reported that despite ustekinumab therapy, her inflammatory bowel disease continued to flare, manifesting as painful bowel movements. Physical examination revealed numerous skin-colored papules, some coalescing into cord-like plaques, of the labia minora, labia majora, and perineum (Fig 1). Dermoscopy revealed multiple round-shaped, yellow structures over a fading pink-brown background (Fig 2). Histological findings of a punch biopsy confirmed the diagnosis (Fig 3).

**Question 1: What is the most likely diagnosis?**

A. Cutaneous CD
B. Eruptive syringomas
C. Fox-Fordyce disease
D. Hidradenoma papilliferum (HPAP)
E. Steatocystoma multiplex (SM)

**Answers:**

A. Cutaneous CD – Incorrect. Cutaneous CD, previously referred to as metastatic CD, is characterized by well-formed granulomatous infiltrates of the skin, discontinuous from the affected gastrointestinal tract. Lesions typically present as ulcers, fistulae, fissures, or abscesses in perianal and orofacial areas.
B. Eruptive syringomas – Correct. Syringomas are benign adnexal tumors of the intradermal portion of the eccrine sweat duct.^1^ A wedge-shaped dermal mass of sweat glands within a noninflamed sclerotic stroma as seen in this case is diagnostic. Eruptive syringomas are a rare variant. Syringomas usually affect the periorbital area of middle-aged women and rarely the genital region.^2^ In a case series of 18 patients with vulvar syringomas, most presented with multiple flesh-colored or brownish papules and less commonly with whitish cystic papules or lichenoid plaques.^2^ While most syringomas are asymptomatic, those on the vulva are often pruritic, as observed in this patient.^2^
C. Fox-Fordyce disease – Incorrect. Fox-Fordyce disease, also called apocrine miliaria, presents with pruritic papules in axillae and anogenital areas.^1^ The pathogenesis involves obstruction of the apocrine gland duct with keratin accumulation in the infundibulum.^1^
D. HPAP – Incorrect. This rare, benign tumor of apocrine origin typically presents as an asymptomatic, skin-colored or reddish nodule in the anogenital region of women aged 30–50.^1^ Unlike syringomas, HPAP does not usually present as multiple papules.^1^
E. SM – Incorrect. SM is an uncommon, benign hamartomatous malformation of the pilosebaceous junction, characterized by multiple asymptomatic cystic papules and nodules. SM rarely involves the vulva. Biopsy may be necessary to differentiate from syringomas.^2^

**Question 2: Which of the following appears to be a risk factor for this condition?**

A. Autoimmune disease
B. Male sex
C. Malignancy
D. Tobacco use
E. Unprotected sex

**Answers:**

A. Autoimmune disease – Correct. Eruptive syringomas have been associated with autoimmune disorders including vitiligo and alopecia areata.^3^ Additionally, the clear cell variant of syringomas is associated with diabetes mellitus. Eruptive vulvar syringomas associated with severe pruritus have been reported in one previous patient with CD.^4^ While the pathophysiology of eruptive syringomas is not completely understood, immunohistochemical staining of syringoma tissue has shown an infiltrate of CD4+ and CD8+ T cells surrounding the sweat ducts.^5^ This suggests that the reactive hyperplasia of cutaneous eccrine ducts may result from autoimmune destruction of their surface components.^5^
B. Male sex – Incorrect. There is an increased incidence of syringomas in females. Onset and/or worsening during puberty, pregnancy, and menstrual cycles suggests that female hormones play a role in their pathophysiology.^3^
C. Malignancy – Incorrect. Syringomas are benign and have not been linked to underlying malignancies. However, they may resemble cutaneous metastases and thus require biopsy for histologic reassurance.
D. Tobacco use – Incorrect. While tobacco use may worsen CD, it is not a reported risk factor for the development of syringomas.
E. Unprotected sex – Incorrect. Syringomas are not sexually transmitted. However, when present on the genitalia, they may resemble condyloma acuminatum or molluscum contagiosum.

**Question 3: Which of these is NOT a treatment option for this condition?**

A. Carbon dioxide (CO2) laser therapy
B. Oral contraceptives
C. Oral isotretinoin
D. Surgical excision
E. Topical atropine

**Answers:**

A. Carbon dioxide (CO2) laser therapy – Incorrect. In a case series of 18 patients with vulvar syringomas, 7 were treated with CO2 laser, resulting in significant relief from pruritus and clinical resolution of lesions.^2^ This treatment modality is particularly beneficial for lesions in sensitive areas due to its precision and ability to minimize scarring. However, patients with skin of color may be at increased risk for postinflammatory hyperpigmentation.
B. Oral contraceptives – Correct. While hormonal influences have been observed, hormonal manipulation is not a recognized treatment. Notably, oral contraceptives have been associated with an increase in size and symptoms of syringomas. Although some investigators have demonstrated estrogen receptor positivity in syringomas, Huang et al did not detect estrogen or progesterone receptors in 15 cases of vulvar syringomas.^2^
C. Oral isotretinoin – Incorrect. Isotretinoin may treat eruptive syringomas by reducing eccrine gland activity.^3^ The drug’s anti-inflammatory properties and ability to inhibit keratinocyte proliferation by modulating epithelial differentiation may also contribute to the observed reduction in size of individual lesions.^3^ Most case reports describe limited efficacy. Topical tretinoin has also been reported to be helpful.^2^
D. Surgical excision – Incorrect. Surgical excision can be used for isolated syringomas. Excision of multiple lesions carries a risk of unacceptable scarring. While removal is generally considered cosmetic, lesions manifesting with severe pruritus may warrant treatment.^4^
E. Topical atropine – Incorrect. Topical atropine has effectively treated eruptive syringomas by alleviating pruritus and reducing lesion size.^2^ Some patients report worsened pruritus and increased lesion size with increased sweating during warmer seasons.^2^

## Conflicts of interest

None disclosed.
